# Evidence of plasticity in *Triodanis perfoliata*: differential flowering response to interannual spring temperature and variation across North America

**DOI:** 10.1093/aobpla/plaf053

**Published:** 2025-09-22

**Authors:** Leidy Laura Arias Martinez, Jennifer J Weber

**Affiliations:** School of Biological Sciences, Southern Illinois University, 1125 Lincoln Drive, Carbondale, IL 62901, United States; School of Biological Sciences, Southern Illinois University, 1125 Lincoln Drive, Carbondale, IL 62901, United States; Evolution & Diversity

**Keywords:** flowering time, phenological sensitivity, phenological shifts, climate change

## Abstract

Examining long-term trends in climate-driven flowering time shifts provides valuable insights, but can mask dynamic interannual variation that may reveal the capacity for short-term phenological responses. We examined the interannual and intraspecific dynamics of flowering time shifts in *Triodanis perfoliata* (Campanulaceae) using a comprehensive dataset with a total 1493 vetted records spanning 1895–2022 across the contiguous USA. Here, we build on previous work demonstrating long-term flowering time advances (Berg et al., An examination of climate-driven flowering-time shifts at large spatial scales over 153 years in a common weedy annual. *Am J Bot* 2019;**106**:1435–43.). Specifically, we examined the influence of interannual temperature variation on flowering time, and explored how these responses varied across a broad geographic range. We found a significant correlation between interannual spring temperature variation and flowering time, with cooler springs associated with delayed flowering and warmer springs associated with earlier flowering. Critically, we found that the magnitude of this relationship varied among *T. perfoliata* populations, with individuals in cooler, higher latitude regions showing less sensitivity to interannual temperature variation than those in warmer, lower latitude regions. This differential sensitivity suggests potential adaptive or plastic responses to local climatic conditions and may have implications for gene flow and the long-term ecological and evolutionary trajectory of *T. perfoliata* populations. This study highlights the importance of considering both long-term trends and interannual variation in phenological research, and emphasizes the need for further investigation into the drivers and consequences of intraspecific variation in phenological sensitivity.

## Teaser

Shifts in flowering time driven by increases in temperature have been documented across a range of flowering plant species. However, intraspecific dynamics of flowering time responses to interannual changes in temperature elucidate the potential for rapid phenological responses. We examine such relationships in a widespread spring flowering annual and show a direct relationship between flowering time and interannual variation in temperature. Further, we demonstrate a spatial pattern in which the sensitivity of these phenological responses varies in relationship to historical local temperatures. In general these data emphasize the importance of exploring phenological trends intraspecifically and across geographic ranges.

## Introduction

Changes in the timing of phenological events are some of the most consistent lines of evidence demonstrating the effects of climate change in natural plant populations (Inouye 2022). Specifically, shifts in flowering time are likely the most well-documented evidence of biological change in the face of climate change (Cleland et al. 2007, Inouye 2022 , Vitasse et al. 2022, Stemkovski et al. 2023). Advances in flowering time with increasing temperatures have been abundantly demonstrated in boreal, temperate, and subtropical regions (Cleland et al. 2007, Iler et al. 2013, Stuble et al. 2021, Vitasse et al. 2022). Such shifts in flowering time may have impacts on community dynamics, such as mismatches in plant–animal interactions, especially between flowering time and pollinator activity (Hegland et al. 2009, Rafferty and Ives 2011, Gérard et al. 2020, Freimuth et al. 2022). Additionally, these shifts may have fitness costs (Scheepens and Stöcklin 2013), or alternatively, facilitate population persistence (Duputié et al. 2015, Mohan et al. 2019). Overall, these evolutionary pressures are likely to be variable both across species and populations (Mohan et al. 2019), leading to potential divergent selection pressure on flowering time between populations (e.g. Hall and Willis 2006, Morente-López et al. 2020). Some of this intraspecific variation is driven by changing patterns of gene flow (e.g. Prevéy et al. 2017), differences in genetic diversity between populations (e.g. Doi et al. 2010) and phenotypic plasticity (Anderson et al. 2012). In addition, these differential responses point to the multifactorial network drivers behind flowering time, such as interactions between multiple abiotic factors that may vary among populations (e.g. photoperiod and temperature; Flynn and Wolkovich 2018, Du et al. 2019, Vorkauf et al. 2021).

Despite considerable work on overall phenological shifts in spring flowering plants, intraspecific variability in phenological sensitivity has been relatively poorly studied. Some studies, however, have demonstrated significant intraspecific variation in phenological sensitivity to temperature across regions (Wang et al. 2015a, Matthews and Mazer 2016, Park et al. 2019, Song et al. 2020, Pearson et al. 2021). For example, intraspecific phenological shifts may vary based on regional variation in precipitation and aridity (Matthews and Mazer 2016, Song et al. 2020) and across longitude and latitude (Wang et al. 2015a). Song et al. (2020) reported that peak flowering time of the widespread perennial herb *Spiranthes sinensis* showed substantial variation in temperature sensitivity, with advances in flowering day of ∼5 days/°C and delays of ∼4 days/°C across 16 humid and nonhumid zones. Because these types of patterns are relatively understudied, the drivers and consequences of differential phenological responses among populations are not well understood.

Long-term trends provide a relatively broad perspective on how species may respond to changing environmental conditions. However, these trends can mask the dynamic and rapid fluctuations that occur over shorter time frames, such as interannual variation. For instance, an advance in flowering time explained by long-term temperature records may be the result of a gradual interannual advance, or alternatively it may be the result of advances and delays in interannual flowering. Understanding interannual variation is essential because it may reveal the ability of species to adapt and respond to more immediate changes in climate, providing evidence of phenological plasticity (Iler et al. 2017). Therefore, a correlation between climatic interannual variation and phenology may indicate plasticity (Anderson et al. 2012). For example, Iler et al. (2013) show this trend, in which long-term trends mask substantial short-term variation in phenological shifts, in two plant communities in snow-dominated ecosystems. Short-term responses can also have direct consequences for plant reproductive success and overall fitness. Iler et al. (2013) found evidence of strong selection for plasticity in flowering time in a subalpine meadow plant community in the face of high interannual variation in abiotic conditions.

**Figure 1.**
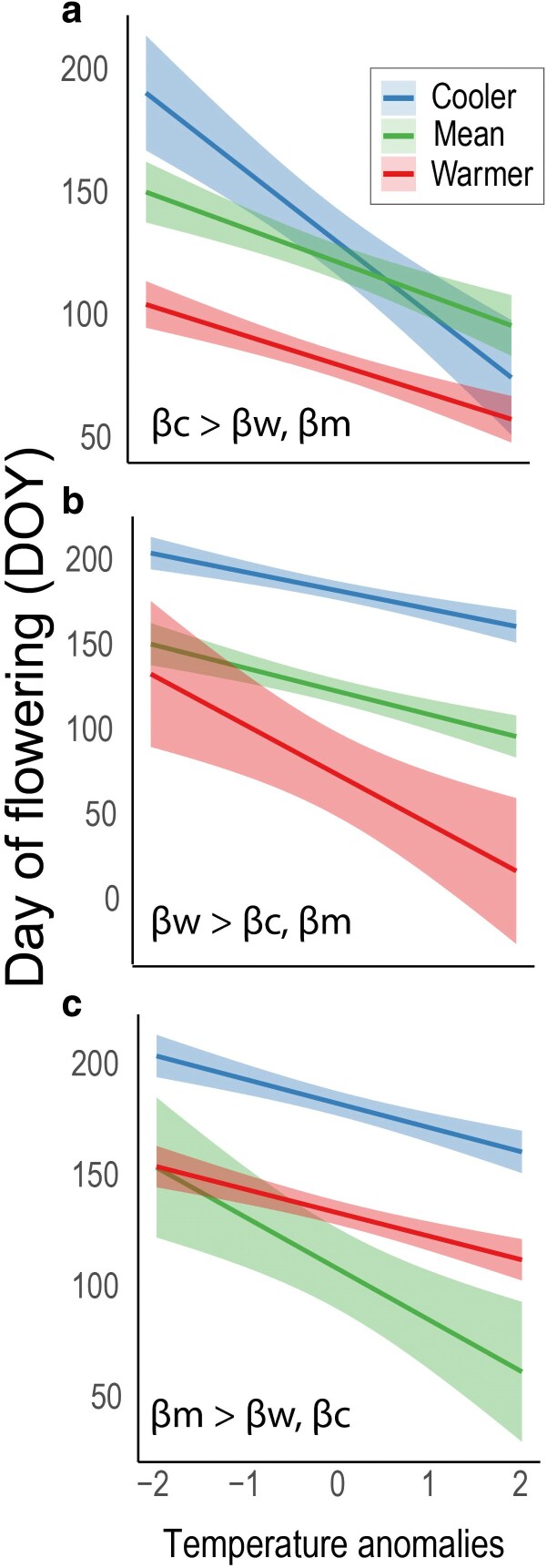
Conceptual diagram of potential patterns of flowering time shifts in relation to local historical temperature. Blue represents localities with relatively cooler historic temperatures (Cooler) with slope βc; red represents localities with relatively warmer temperatures (Warmer) with slope βw, and green represents localities with historically intermediate temperatures (Mean) with slope βm. a) Higher phenological sensitivity to changes in interannual temperature (Temperature anomalies) in localities with relatively lower historic temperatures. b) Higher phenological sensitivity in localities with relatively higher temperatures. c) Higher phenological sensitivity in localities with historically intermediate temperatures.

Long-term trends, on the other hand, offer a more general perspective, representing the cumulative effects of climate change over extended periods, but may not reveal the intricate dynamics that occur within shorter time periods. By studying interannual variation and how it varies geographically, we gain a clearer picture of how species and populations respond to climate variability. Overall these processes can result in dynamic interactions, where different short-term responses between populations may be associated with local historical climatic conditions (e.g. mean annual temperature; Fig. 1). For instance, populations at localities with lower than average local temperatures may exhibit relatively high sensitivity to changes in interannual temperature (Fig. 1a); in these populations, selection may favour relatively short phenological cycles and high sensitivity to rapid climatic changes. In contrast, populations in areas with relatively higher temperatures may exhibit heightened phenological sensitivity (Fig. 1b). Such a pattern could be predicted if the climate in these areas is relatively stable (associated with lower latitudes). In such environments, the benefits of closely tracking spring temperature cues are greater, as the risk of late-spring frost damage is reduced, resulting in reduced risk for early flowering (Miller et al. 2023). These different population sensitivities may lead to different evolutionary and ecological outcomes, with implications for the way in which populations respond to climate change. The use of a widespread species as a study system is fundamental to better understand these potential patterns in phenological sensitivity to interannual climate change. Using *Triodanis perfoliata* (Campanulaceae) as a study system, we take advantage of a large, thorough dataset built using digitized herbarium resources as well as citizen science data, to better understand potential patterns of flowering time shifts, especially for widespread species.

In this study, we examine the fine-scale dynamics of phenological shifts, with a focus on flowering time among populations of *T. perfoliata*. Long-term analyses revealed that this species has advanced its flowering time in association with an increase in spring temperature (Berg et al. 2019). Here we examine much finer scale temporal and spatial patterns in the magnitude and patterns of flowering time shifts for this species. Using digitized herbarium and citizen science we encompassed 127 years of spatiotemporally explicit flowering time and climatic data. Given the broad distribution of this species, we hypothesized that *T. perfoliata* would exhibit intraspecific variation in phenological sensitivity to interannual variation (Fig. 1). To test this, we examined flowering time responses to interannual variation in abiotic factors (e.g. temperature), and described how these patterns varied across the sampled geographic range (i.e. the contiguous USA). We also determined if *T. perfoliata* showed a pattern of rapid flowering time shifts in response to interannual climatic change, and whether this response varied according to local historical temperatures.

## Materials and methods

### Study system

*Triodanis perfoliata* (L.) Nieuwl. (Campanulaceae) is a common, widespread annual plant that grows readily in disturbed habitats such as roadsides, trails and agricultural fields (Weakley 2010). The distribution spans from much of the USA and Mexico through South America. In the USA, flowering time is restricted to the early spring season (Berg et al. 2019, 2023) and it is recognized for its purple, five-petaled flower (Trent 1940, Weakley 2010). This species exhibits dimorphic cleistogamy, including open self-compatible flowers (chasmogamous) and closed, obligate selfing flowers (cleistogamous) (Trent 1940). Both types of flowers occur simultaneously in the last two thirds of the flowering period (Trent 1940). Across the geographic range within the contiguous USA, the peak flowering time for this species has advanced approximately 9 days from 1863 to 2016 (Berg et al. 2019).

### Phenological and climate data collection

An original dataset from Berg et al. (2019) was included and significantly developed with additional data points using the same criteria. Briefly, for Berg et al. (2019), online portals of 19 herbarium networks and iNaturalist (www.inaturalist.org) were used; the flowering dates were determined based on the presence of chasmogamous flowers which typically occur in the middle of the flowering window (Trent 1940), indicating ‘peak flowering’. We updated the phenological dataset of Berg et al. (2019) for *T. perfoliata* in the USA to include more contemporary records, and to supplement years that included fewer than ten ‘peak flowering’ records in the original dataset. We were able to accomplish this because more digitized herbarium records are now available since the publication of Berg et al. (2019). We used digitized herbarium collections (Supplementary Table S1), including iDigBio (www.idigbio.org), the Intermountain Region Herbarium Network (www.intermountainbiota.org), and iNaturalist. In this study, we examined only records that indicated open flowers (i.e. peak flowering), similar to the Berg et al. (2019) study, however the entire dataset (See Data Availability) also includes data for ‘vegetative’, ‘fructification’, and ‘senescence’, but these phenophases represented a relatively small dataset, and were not examined as part of our current research. Phenophases were determined by visual inspection of the specimen when it was available, or label transcription was used when the image was not available. A total of 529 new records were added to the original data set. We vetted our new dataset for accuracy by removing data points with nonsensical georeferences (e.g. localities in bodies of water). A final combined dataset (with the Berg et al. 2019 dataset) of 1649 records was obtained. This entire dataset was then vetted to reduce spatial bias (i.e. records that were relatively close to each other); this was performed within a 20 km radius for each month-year combination, resulting in a final dataset of 1493 records spanning 1895 to 2022 in the USA. Also, this resulted in a final dataset with at least ten records per year for peak flowering for *T. perfoliata* from 1895 to 2022 across the contiguous USA (See Data Availability).

Berg et al. (2019) found that variation in flowering time was explained best by variation in temperature; therefore, for this study we focused on variation in temperature as primary abiotic cue for peak flowering (Berg et al. 2019). Annual mean temperature data from 1895 to 2022 and monthly mean temperature from 1895 to 2022 were extracted for each spatially and temporally explicit record using the PRISM dataset (https://prism.oregonstate.edu), with 4 km of resolution.

### Flowering response to interannual temperature variability and intraspecific variability

An initial analysis was performed to determine which temperature month explained most of the variation in peak flowering time (DOY = day of year) to reduce model complexity; generalized linear models (GLM) were used to examine the relationship between DOY for flowering and each month's mean temperature. Across the broader dataset and consistent with the findings of Berg et al. (2019), spring temperatures in March, April, and May (MAM) significantly explained the overall variation in DOY (Supplementary Table S2). Subsequently, variation in spring temperatures (mean temperatures for MAM) were used for examination of interannual flowering time responses in all analyses.

#### Estimation of temperature anomalies

Following the approach implemented by Pearson et al. (2021), interannual temperature variation (e.g. temperature anomaly) was estimated as the difference between the mean spring temperature (mean temperatures for MAM) in the year of each record (e.g. grid cell) and the long-term mean (1895–2022) spring temperature for that spatially explicit record. Hereafter, we refer to this calculation of interannual temperature variation as Mean Anomalies Spring = MeAnSpring.

#### Estimation of local historical temperatures

Next, we examined how interannual variability in flowering time responses might be influenced by local historical temperatures (intraspecific variability). The average of annual temperatures from 1895 to 2022 for each occurrence (MAT hereafter) provides a historical and site specific estimate of long-term temperatures per locality. To translate this into a geographic variation, we categorized MAT into three levels (using intervals Sturges’ rule); localities that were cooler than the annual average historical temperatures (Cooler), 1.74°C–12.39°C, localities with exhibited approximately the average annual temperatures (Mean), 12.40°C–16.22°C, and localities with warmer than the average temperatures (Warmer), 16.23°C–23.33°C.

#### Statistical analysis

The relationship between peak flowering (DOY), the MeAnSpring and the level of MAT was examined using GLMs with both negative binomial and Poisson distributions (as appropriate for count data). The *lme4* (Bates et al. 2015), *car* (Fox and Weisberg 2018), and *MASS* (Venables and Ripley 2002) packages in R were used for all analyses. A *post hoc* multiple comparison test between the MAT levels was conducted using *emmeans* (Lenth 2025). The DOY for flowering may be significantly explained by interannual variation (indicated by an significative relationship of MeAnSpring with DOY in the model), it may also be associated with the local historical temperatures (indicated by an significative relationship of MAT levels with DOY), and may be explained by the interaction between MAT levels and MeAnSpring. Thus, the final global model was DOY ∼ MeAnSpring + MAT levels + MeAnSpring * MAT levels.

## Results

**Figure 2.**
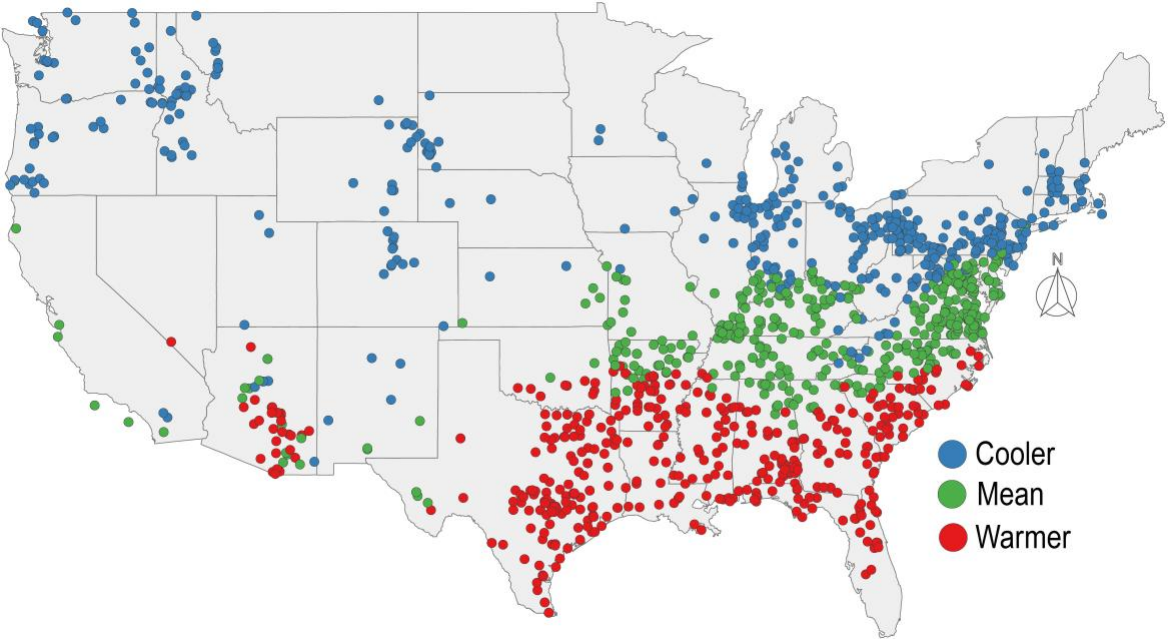
Spatial distribution of MAT (mean annual temperatures from 1895 to 2022) levels (*n* = 1493) for each spatially explicit record of flowering time for *T. perfoliata*. Blue represents localities with relatively cooler historic temperatures (Cooler); red represents localities with relatively warmer temperatures (Warmer), and green represents localities with historically intermediate temperatures (Mean).

MAT levels showed a predictable distinct pattern across the geographical area addressed in this study. First, records of *T. perfoliata* with local historical temperatures ranging from 1.74°C to 12.39°C (MAT level = Cooler) were mainly concentrated in the northern USA and in the higher latitudes of the northwestern USA (Fig. 2); records in the ‘Mean’ MAT level (12.40°C–16.22°C) were concentrated in the mid-east USA. Finally, records from localities in the ‘Warmer’ MAT level, were prevalent in the south-east of the USA, with some records in the south-west (Fig. 2).

### Flowering response to interannual temperature variability and intraspecific variability

The response variable (DOY of peak flowering) was not best described by a normal distribution (Shapiro–Wilk test *P*-value < 0.05); two additional probability distributions were examined (negative binomial and Poisson). A negative binomial distribution best described the GLM, using the Akaike and log likelihood criteria, therefore, posterior models were based exclusively on a negative binomial distribution.

#### Flowering time and temperature anomalies

**Figure 3.**
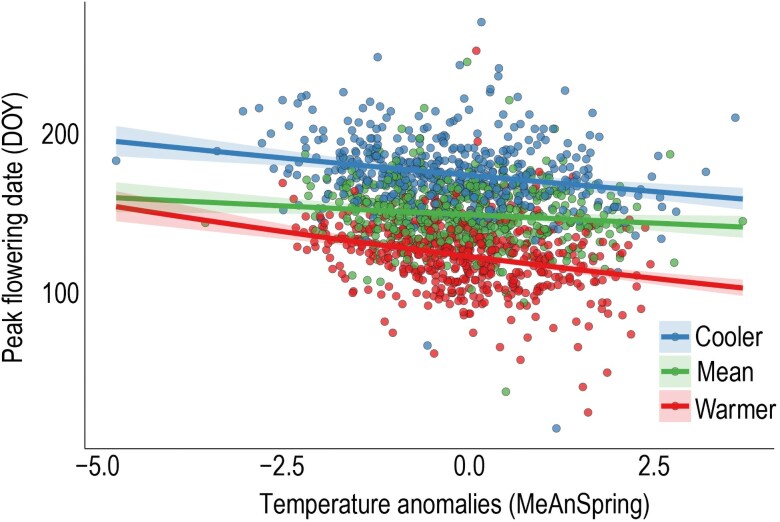
The relationship between peak flowering date (DOY) and temperature anomalies (MeAnSpring) (*n* = 1493; LR chisq = 66.0, *P*-value = 4.4e−16), at three different levels of historical spring temperatures (MAT levels; LR Chisq = 1488.5, *P*-value = 2.2e−16). Blue represents localities with relatively cooler historic temperatures (Cooler); red represents localities with relatively warmer temperatures (Warmer), and green represents localities with historically intermediate temperatures (Mean). Shaded areas around each trend line indicate 95% confidence intervals.

Overall, interannual variation for mean spring temperatures was significantly correlated to DOY of peak flowering (LR Chisq = 66.0, *P*-value = 4.4e−16, Supplementary Table S3), with negative interannual variation in spring temperature (cooler springs) associated with later flowering; and positive interannual variation (warmer springs) associated with earlier flowering (Fig. 3).

#### Flowering time and local historical temperatures

Peak flowering date (DOY) was also explained significantly by local historical temperatures (MAT levels; LR Chisq = 1488.5, *P*-value = 2.2e−16). The magnitude of flowering response between the three historical temperature levels (Cooler, Mean, and Warmer) varied significantly. Specifically, localities with warmer local historical temperatures, exhibited greater flowering date (DOY) sensitivity to the change in interannual spring temperature variation (i.e. slope) than cooler localities (z-ratio = 2.8, *P*-value = 1.0e−2), and localities with mean local historical temperatures (z-ratio = 3.7, *P*-value = 7.0e−4). For each one-unit increase in MeanSpring, the change in peak flowering date at warmer localities was 0.02 greater than at cooler localities; and 0.03 greater than at localities exhibiting mean annual historical temperatures (Supplementary Table S4). There was no significant difference in the magnitude of the flowering time response between ‘Cooler’ and ‘Mean’ localities (z-ratio = −1.2, *P*-value = 0.5) (Supplementary Table S4). For *T. perfoliata* populations sensitivity to interannual changes in spring temperature follows a model of higher sensitivity at localities with relatively higher historical local temperatures (Fig. 1b).

Overall, the interaction between MeAnSpring and MAT levels was also highly significant (LR Chisq = 14.4, *P*-value = 7.4e−4), suggesting DOY is dependent on the interaction between the interannual spring variation (e.g. anomalies) and the local historical temperatures from 1895 to 2022.

## Discussion

Flowering time and other phenological responses (e.g. leaf emergence) to interannual climate change have been found in a range of plants and in different biomes (Cayan et al. 2001, Munson and Long 2017, Zheng et al. 2018, Ramirez-Parada et al. 2022). Our data is consistent with these studies, showing a short-term flowering response to changes in interannual spring temperatures. In *T. perfoliata*, the average day of peak flowering is associated with interannual spring temperature variation, but the magnitude of this relationship depends on the population locality. Specifically, *T. perfoliata* exhibits a relatively later peak flowering date in cooler localities and an earlier flowering date in warmer localities. Our results support previous studies (Berg et al. 2019) that have shown the advancement of peak flowering day in relation to spring temperature increase for much of the range of *T. perfoliata*. Taken together, these previous studies and our results emphasize the need to include multiple parameters in predictive models, as focusing solely on long-term trends may overlook significant phenological responses to interannual temperature variation. Across species, understanding these short-term responses is crucial for accurately predicting how plant phenology will shift under ongoing climate change.

### Short-term and differential flowering response in *T. perfoliata* across the USA

The examination of interannual variation within long-term climatic trends may reveal the direct relationship between phenology and climatic variables (Anderson et al. 2012, Iler et al. 2017). Our results indicate an initial sign of plasticity in flowering time in *T. perfoliata* and suggest that plasticity in this species may be beneficial for phenological tracking of short-term climatic fluctuations. However, even if plasticity allows for short-term phenological shifts, it does not guarantee the long-term ability to track climate change. Ongoing climate change may impart environmental conditions outside known historical variability, or move beyond species physiological limitations (Anderson et al. 2012). Also see Ghalambor et al. 2007, Fox et al. 2019).

Here, we demonstrate that relatively earlier flowering is associated with warmer springs compared to relatively delayed flowering associated with cooler springs. Contrasted with long-term trends, this is a relatively rapid flowering response, and such a response may have important ecological implications. For example, rapid shifts in flowering time may increase the likelihood of mismatches in local biological interactions. For *T. perfoliata*, which is largely pollinated by generalists such as small bees and flies (Tillotson 2023), shifts in the timing of open, chasmogamous flowers may disrupt pollinator mutualisms. Previous work in this species by Ansaldi et al. (2018) has also demonstrated that pollination environments can alter allocation to closed, obligately selfing flowers relative to open flowers. Therefore, a decrease in pollinator activity or a change in the quality of pollination services has the potential to influence the breeding system of *T. perfoliata*, leading to changes in the extent of self-fertilization. Future research using experimental approaches would be valuable to determine the specific conditions under which these short-term responses are adaptive and how they interact with broader climate trends.

Intraspecific variation in phenological sensitivity among different geographic regions is also supported by our data. We found that individuals from relatively warmer localities advanced their flowering day more rapidly in response to interannual temperature changes, whereas individuals from cooler localities (concentrated in the highest latitudes) required larger interannual temperature changes to advance flowering. Evidence for this trend in sensitivity is indicated by differences in the slopes among records originating from localities with different historical local temperatures. Plants in colder areas may be adapted to more variable interannual temperature conditions (e.g. greater local spring temperature variance) (Pau et al. 2011) and therefore have low temperature sensitivity (Zhang et al. 2015). Similarly, at high latitudes, rapid within-spring warming has been associated with this low temperature flowering sensitivity (Wang et al. 2015b, 2016). Overall, this suggests that in these areas at higher latitudes, the lack of gradual increases in spring temperature may have shaped flowering sensitivity to temperature changes in *T. perfoliata* populations in a plastic or adaptive manner, with a possible reduced ability to track long-term increases in spring flowering sensitivity due to climate change. Such patterns could have consequences for the overall flowering ecology within these populations.

As in our study, Zhang et al. (2015) found that spring plants tend to be more sensitive to small changes in temperatures, and exhibit flowering day shifts in warmer areas. However, another pattern has been observed for high latitude tundra plant communities, with a more sensitive flowering response to increase in summer temperatures at colder relative to warmer locations (Prevéy et al. 2017). Differences in longitudinal scales, geographical factors and seasonal patterns possibly associated with life history traits may reflect the multidimensional fixed factors influencing phenological sensitivity to temperature changes. These and our results showed variability in how rapidly different populations respond to temperature changes relative to geography and historical climates.

The pattern found for *T. perfoliata* populations best fit the model which explicitly showed differences in phenological sensitivity in terms of differences in the historical regimes of the localities (Fig. 1b). The observed differences in flowering sensitivity across the sampled distribution of *T. perfoliata* indicate the different ecological and evolutionary trajectories across populations. Several implications, such as limitations to gene flow, may be expected as an effect of this intraspecific variation (e.g. divergent flowering times limiting gene flow or pollinator efficacy). Specifically, gene flow may be restricted by asynchronous flowering between populations, with successful migration between populations with similar flowering times (Weis 2015), which may lead to reproductive isolation barriers between populations with different historical climate patterns (e.g. Hall and Willis 2006). Intraspecific variation in flowering sensitivity may also impact population-specific interactions with competitors or herbivores (Love and Mazer 2021). In summary, the difference in phenological sensitivity across the distribution of *T. perfoliata* (and other similar annual plants) may impact gene flow, and also create substantial variability in ecological interactions, resulting in divergent evolutionary trajectories. Future studies exploring genetic structure in relation to flowering time and phenological sensitivity are needed to create a better understanding of these patterns and the capacity for *T. perfoliata* populations to track climate change. In addition, using experimental approaches in controlled conditions would allow for quantification of the plastic and genetic basis for flowering time among populations of *T. perfoliata*, as well as allow for an estimate of the fitness consequences of shifting flowering times.

For the genus *Triodanis*, this is the first record of intraspecific variation in flowering time associated with local historical climatic conditions. Our findings complement and build on those of Berg et al. (2019). Our study refines our understanding of flowering time shifts in *T. perfoliata* by highlighting the role of intraspecific patterns in flowering time sensitivity as well as responses to interannual variation. In the broader context, our data combined highlights the many factors that influence the capacity for phenological tracking as well as the patterns driven by these factors.

## Supplementary Material

plaf053_Supplementary_Data

## Data Availability

Raw data and R code are available online at: https://github.com/leidylauraariasmartinez/TriodanisFloweringTime.
