# Supplementary material for: Evidence of plasticity in Triodanis perfoliata: differential flowering response to interannual spring temperature and variation across North America
Source: AoB Plants. 2025 Sep 22;17(5):plaf053. doi: 10.1093/aobpla/plaf053 (PMC12516698; doi:10.1093/aobpla/plaf053)
Supplement: plaf053_Supplementary_Data [file plaf053_supplementary_data.docx]

**Supplementary material**

Table S1**:** List of resources used for digitized records indicating spatially and temporally explicit phenological data for *Triodanis perfoliata* in this study.

| **Source** | **Type** | **url** |
| --- | --- | --- |
| iNaturalist | Citizen science | https://www.inaturalist.org/ |
| iDigBio | Coordinating center for the national digitization effort | https://www.idigbio.org/ |
| Intermountain Regional Herbarium Network | Herbaria network | https://intermountainbiota.org/portal/ |

Table S2: Generalized linear model for peak flowering time (DOY) in terms of the mean temperature for each month from 1895 to 2022 (using a negative binomial distribution). The best predictive months for DOY were March, April and May, with a p-value of less than 0.001, consistent with Berg *et al*. 2019.

| **Factor** | **Estimate** | **Std. Error** | **z value** | **Pr(>\|z\|)** |
| --- | --- | --- | --- | --- |
| TmJanuary | -1.410e-03 | 1.244e-03 | -1.133 | 0.25703 |
| TmFebruary | -3.440e-03 | 1.301e-03 | -2.643 | 0.00821 |
| TmMarch | -6.607e-03 | 1.438e-03 | -4.594 | 4.34e-06 |
| TmApril | -1.082e-02 | 2.031e-03 | -5.330 | 9.81e-08 |
| TmMay | -1.030e-02 | 2.243e-03 | -4.590 | 4.43e-06 |
| TmJune | -3.692e-03 | 2.596e-03 | -1.422 | 0.1549 |
| TmJuly | 6.112e-03 | 2.913e-03 | 2.098 | 0.03592 |
| TmAugust | 4.828e-05 | 2.858e-03 | 0.017 | 0.98652 |
| TmSeptember | -1.706e-03 | 2.537e-03 | -0.673 | 0.50125 |
| TmOctober | -2.747e-03 | 2.066e-03 | -1.330 | 0.18356 |
| TmNovember | 4.404e-04 | 1.731e-03 | 0.254 | 0.79919 |
| TmDecember | -1.810e-03 | 1.360e-03 | -1.330 | 0.18340 |

Table S3: Likelihood Ratio Tests (for the binomial negative model) explaining peak flowering date (DOY) in relation to spring anomalies (MeAnSpring), the local historical temperatures (MAT level) and the interaction between them (MeAnSpring* MAT level).

| **Factor** | **LR Chisq** | **Df** | **Pr(>Chisq)** |
| --- | --- | --- | --- |
| MeAnSpring | 66.03 | 1 | 4.43e-16 |
| MAT level | 1488.52 | 2 | < 2.2e-16 |
| MeAnSpring* MAT level | 14.40 | 2 | 0.0007461 |

Table S4: Estimated marginal trends of each local historical temperature category (MAT level) and Tukey-adjusted pairwise comparisons.

| **MAT level / Contrast** | **Estimated slope** | **z.ratio** | **p.value** |
| --- | --- | --- | --- |
| Cooler | -0.0243 | —-- | —-- |
| Mean | -0.0145 | —-- | —-- |
| Warmer | -0.0478 | —-- | —-- |
| Cooler - Mean | —-- | -1.182 | 0.4640 |
| Cooler - Mean | —-- | 2.803 | 0.0140 |
| Mean - Warmer | —-- | 3.677 | 0.0007 |
